# Predicting Survival of Patients With Rectal Neuroendocrine Tumors Using Machine Learning: A SEER-Based Population Study

**DOI:** 10.3389/fsurg.2021.745220

**Published:** 2021-11-03

**Authors:** Xiaoyun Cheng, Jinzhang Li, Tianming Xu, Kemin Li, Jingnan Li

**Affiliations:** ^1^Department of Gastroenterology, Peking Union Medical College Hospital, Chinese Academy of Medical Sciences and Peking Union Medical College, Beijing, China; ^2^Key Laboratory of Gut Microbiota Translational Medicine Research, Peking Union Medical College Hospital, Chinese Academy of Medical Sciences and Peking Union Medical College, Beijing, China; ^3^Department of Physiology and Pathophysiology, School of Basic Medical Sciences, Capital Medical University, Beijing, China

**Keywords:** rectal neuroendocrine tumors, machine learning, predictive model, SEER database, rectal cancer

## Abstract

**Background:** The number of patients diagnosed with rectal neuroendocrine tumors (R-NETs) is increasing year by year. An integrated survival predictive model is required to predict the prognosis of R-NETs. The present study is aimed at exploring epidemiological characteristics of R-NETs based on a retrospective study from the Surveillance, Epidemiology, and End Results (SEER) database and predicting survival of R-NETs with machine learning.

**Methods:** Data of patients with R-NETs were extracted from the SEER database (2000–2017), and data were also retrospectively collected from a single medical center in China. The main outcome measure was the 5-year survival status. Risk factors affecting survival were analyzed by Cox regression analysis, and six common machine learning algorithms were chosen to build the predictive models. Data from the SEER database were divided into a training set and an internal validation set according to the year 2010 as a time point. Data from China were chosen as an external validation set. The best machine learning predictive model was compared with the American Joint Committee on Cancer (AJCC) seventh staging system to evaluate its predictive performance in the internal validation dataset and external validation dataset.

**Results:** A total of 10,580 patients from the SEER database and 68 patients from a single medical center were included in the analysis. Age, gender, race, histologic type, tumor size, tumor number, summary stage, and surgical treatment were risk factors affecting survival status. After the adjustment of parameters and algorithms comparison, the predictive model using the eXtreme Gradient Boosting (XGBoost) algorithm had the best predictive performance in the training set [area under the curve (AUC) = 0.87, 95%CI: 0.86–0.88]. In the internal validation, the predictive ability of XGBoost was better than that of the AJCC seventh staging system (AUC: 0.90 vs. 0.78). In the external validation, the XGBoost predictive model (AUC = 0.89) performed better than the AJCC seventh staging system (AUC = 0.83).

**Conclusions:** The XGBoost algorithm had better predictive power than the AJCC seventh staging system, which had a potential value of the clinical application.

## Introduction

Gastroenteropancreatic neuroendocrine tumors (GEP-NETs) are heterogeneous malignancies that originate from gastrointestinal peptidergic neurons and neuroendocrine cells. The rectum is one of the most common primary sites of GEP-NETs. As mass screening for gastrointestinal cancer has become more widespread and endoscopy has advanced, the number of patients diagnosed with rectal neuroendocrine tumors (R-NETs) is increasing year by year. In the United States, the Surveillance, Epidemiology, and End Results (SEER) registry database show that the incidence of R-NETs increased from 0.2 per 100,000 in 1973 to 0.86 per 100,000 in 2004 (1). Although the 5-year survival is high overall (range, 83%−94%), patients with nodal disease and distant metastases have a poor prognosis and high rates of mortality (2). Early study based on the SEER estimated the 5-year survival of localized, regional, and distant metastatic R-NETs were 90, 62, and 24%, respectively (3). Therefore, effective models to predict the prognosis of R-NETs are required.

Tumor–node–metastasis (TNM) staging system proposed by the American Joint Committee on Cancer (AJCC) and the European Neuroendocrine Tumor Society (ENETS) and pathology classification proposed by the World Health Organization have been considered as prognostic systems of R-NETs. However, these systems mainly include tumor size, organ invasion, mitotic count, and Ki-67 as predictors (1, 4, 5). Many other factors, such as age, sex, tumor numbers, or treatment, are not involved. Previous studies are heterogeneous when the respective roles of staging and grading are compared (6), so an integrated survival predictive model composed of various clinicopathological characteristics is immediately needed.

Recently, the use of artificial intelligence for medicine has drawn much attention, especially in model data. In artificial intelligence, machine learning can help analyze implicit useful information from a large number of data and reveal the relationship between data. Multiple machine learning algorithms could be used in building predictive models.

The present study is aimed at exploring epidemiological characteristics of R-NETs based on a retrospective study from the SEER database and predicting the survival of R-NETs with machine learning algorithms.

## Materials and Methods

### Participants

For this research, data of patients with R-NETs were extracted from the SEER database (2000–2017), using the SEER^*^ Stat software version 8.3.6.1. The primary site code (C20.9, rectum, NOS) and the following International Classification of Diseases for Oncology, Third Edition (ICD-O-3), histology codes were used to identify cases with R-NETs: 8240 (Carcinoid tumor), 8246 (Neuroendocrine carcinoma), and 8249 (Atypical carcinoid tumor). We included cases with cancer diagnosed microscopically and excluded cases with cancer diagnosed solely by autopsy or death certificate.

Data including age at diagnosis, sex, race, grade, tumor size, survival months, distant metastasis, surgery type, AJCC seventh stage, death status were retrieved from the SEER database. The main outcome measure was overall survival. In addition, we collected data of patients with R-NETs in Peking Union Medical College Hospital from January 2012 to January 2016. The same encoding was adopted as the SEER database to validate the predictive model. Patients with missing data were excluded. Since the predictive model needed to classify the data, we chose the 5-year survival status as the target of the predictive model. The patients included in the study were who died during the 5-year follow-up and survived for >5-year follow-up period. Because the follow-up period was not long enough to obtain an accurate 5-year survival status, we excluded patients who survived for <5-year follow-up period.

### Data Preprocessing

In this study, data were preprocessed according to the characteristics of the SEER database. For the text record, we used label encode to convert it into numerical values to facilitate data processing by machine learning algorithms. For unordered categorical variables with three or more categories, we used one hot encode to convert them into multiple binary categorical variables, so the data could be used more effectively.

For the sake of interpretability, we simplified partial data. The primary site surgery was simplified to tumor destruction, tumor resection, no surgery, and an unknown type of surgery. In addition, the data of tumor size were simplified to tumor size larger than 1 cm or not. For the missing data in the database, we calculated the Euclidean distance between each case and used the mean values of the five closest cases to estimate and fill in the missing values. This method is called the k-Nearest Neighbors approach (7).

For most machine learning algorithms, when variables range from 0 to 1, the optimum conditions are obtained. Data were scaled to the range from 0 to 1 at the preprocessing stage to boost the efficiency of machine learning algorithms.

### Establishment of Predictive Model

We used Python's scikit-learn 0.24.1 package to construct a machine learning predictive model (8). The scikit-learn 0.24.1 package is widely used because it includes common machine learning algorithms. In this study, we selected six common machine learning algorithms to build predictive models. Among the support vector machine algorithms, we chose the C-Support Vector Classification (SVC) and Nu-SVC with the radial basis function kernel. Among the ensemble-based algorithms, we chose the random forest (RF) algorithm, AdaBoost algorithm, and eXtreme Gradient Boosting (XGBoost) algorithm. The Naive Bayes (NB) algorithm is also considered to have a good predictive ability, so we also chose it to build a predictive model. For comparison, we chose the AJCC seventh staging system as a representative of clinically used predictive models.

### Feature Selection

First, we used all the demographic characteristics, tumor grade, tumor size, tumor metastasis, and other descriptive information obtained from the SEER database as the features to optimize the predictive power of the machine learning models. We used the machine learning algorithms described above to establish preliminary predictive models under default conditions and used Shapley additive explanations (SHAP) to assess the importance of each feature. SHAP is a method of interpreting machine learning predictive models. It can analyze the impact of each feature of each patient on the predictive results (9). Integrating the SHAP results of all preliminary predictive models, features that are considered important in all preliminary predictive models are used as the final features of the machine learning model.

### Ten-Fold Cross-Validation

Ten-fold cross-validation is a commonly used method to evaluate predictive capacity in the process of constructing machine learning predictive models. Due to the characteristics of machine learning algorithms, the data used in the training process cannot be used to test the trained predictive model again. Therefore, when we evaluated machine learning predictive models, the data from the SEER database were divided into training data and test data. In the 10-fold cross-validation process, we equally divided the data into 10 sets, from where one set was selected as training data and the remaining 9 were testing data. The training data were used to train machine learning predictive models, and then the testing data were used to calculate the receiver operating characteristic (ROC) curve and area under the curve (AUC) of the predictive model. The results obtained with 10 repetitions and the average ROC curve over the generated 10 different ROC curves were employed to assess the performance of the predictive model.

### Parameter Adjustment

The parameter settings of the machine learning algorithms will affect the predictive ability of the predictive model and each machine learning classifier has different parameter settings. In the present study, we used the grid-search algorithm to determine the optimal parameters for each machine learning algorithm.

We used the potential range of optimal parameters to establish the predictive model one by one and used 10-fold cross-validation to compare the predictive ability of the predictive model relying on parameter combinations. Then we selected parameter combinations with the best predictive capacity. This process is called the grid-search algorithm. By changing the ranges of parameters, the best parameter combination of the machine learning algorithm was finally obtained.

### Evaluation of Predictive Models

The predictive power built by six machine learning algorithms was assessed by a 10-fold cross-validation procedure. For comparisons based on the ROC curves and the AUC, we selected the predictive model with the best predictive power as the representative of the machine learning predictive model. To compare with traditional predictive models, we used the AJCC seventh staging system as the representative of traditional predictive models. The data from the SEER database for the period 2000–2009 were used as the training set, and the data from the SEER database for the period 2010–2017 were used as the internal validation set. In the internal validation set, we also deleted patients with missing AJCC staging. External validation with a cohort from Peking Union Medical College Hospital was performed. The training set was used to train the machine learning predictive model. We calculated the ROC curve and the AUC of predictive models using the internal validation set and the external validation set for internal and external validation. We compared the best machine learning predictive model with the AJCC seventh staging system to evaluate their predictive performance. In addition, we used the parametric approach based on Platt's logistic model to calibrate model-predicted probabilities and calculated the Brier score to evaluate the calibration performance of the predictive model.

### Statistical Analysis

R 4.0.4 was used for data description and statistical analysis. According to whether continuous variables satisfied the normal distribution, data expressed as mean ± standard deviation or median, first, and third quartiles. We used Student's *t*-test or the Mann–Whitney *U* test for statistical analysis. Categorical variables were expressed by frequency (*n*) and percentage (%). We conducted Chi-square test or Fisher's exact test for statistical analysis. Net reclassification index (NRI) was deployed to compare the performance improvement of the predictive model. *P*-value of < 0.05 was considered statistically significant, and all *P* values were two sided.

## Results

### Patient Characteristics

**Figure 1:**
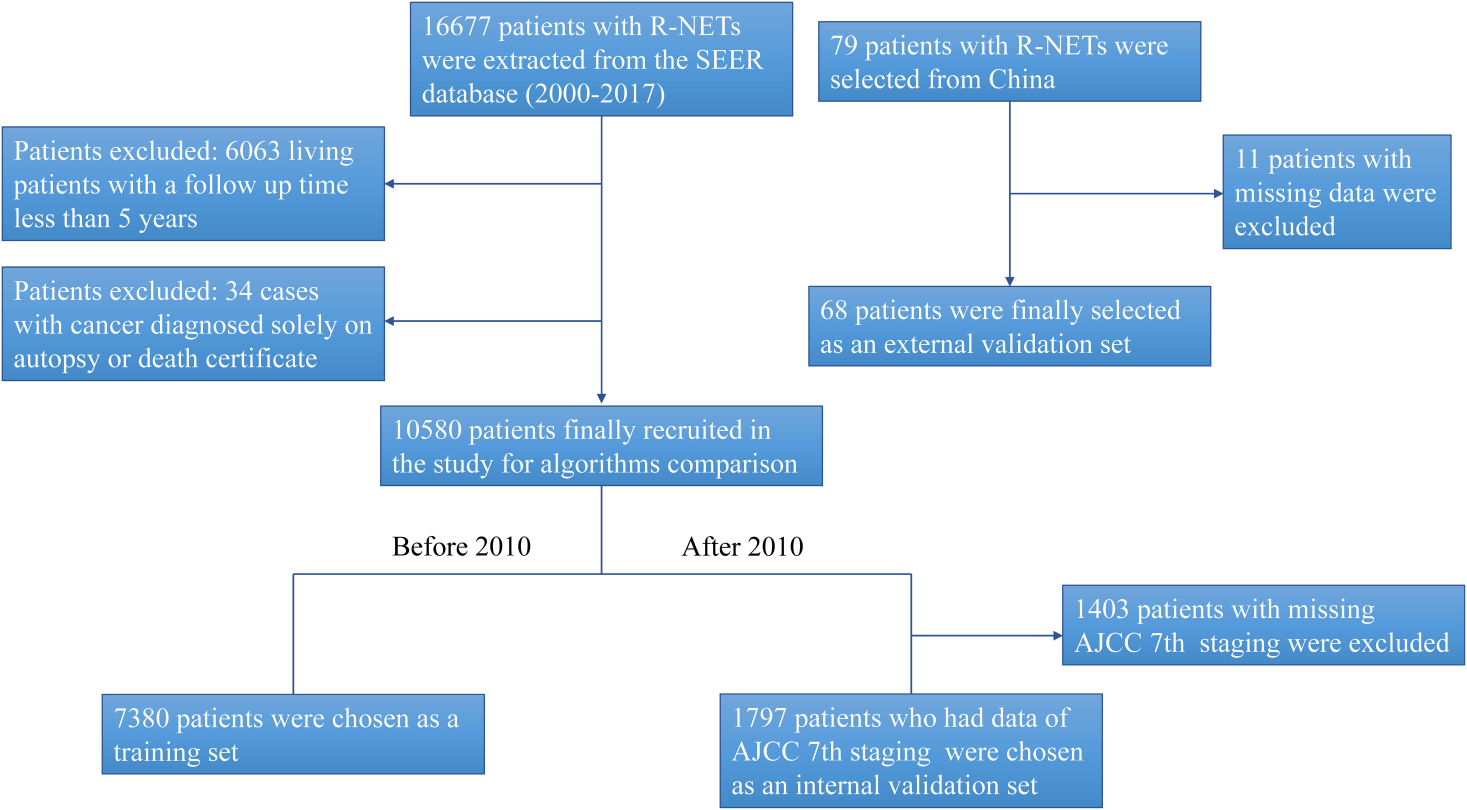
The flow chart for study identification, screening, and inclusion in the training set and validation set.

A total of 16,677 patients with R-NETs were extracted from the SEER database and a total of 79 patients with R-NETs were collected from Peking Union Medical College Hospital according to the inclusion criteria. Based on the exclusion criteria, a total of 10,580 patients from the SEER database and 68 patients from Peking Union Medical College Hospital were finally included in the analysis (Figure 1). Basic characteristics of patients from the SEER database were summarized in Table 1, the median age of the study group was 56.3 years old and 49.8% were male. Many baseline characteristics were significantly different between the patients who survived for more than 5-years of the follow-up period and patients who died during the 5-year follow-up period.

**Table 1:** Main characteristics of the patients.

**Variable**	**Overall**	**Survived for more than 5-year follow-up**	**Died during the 5-year follow-up**	***P*-value**
Age (years)	56.3 ± 11.9	55.1 ± 11.2	64.5 ± 13.0	<0.001
Gender (%)				<0.001
Male	5,267 (49.8%)	4,418 (48.1%)	849 (60.8%)	
Female	5,313 (50.2%)	4,765 (51.9%)	548 (39.2%)	
Race (%)				<0.001
White	6,099 (57.6%)	5,280 (57.5%)	819 (58.6%)	
Black	2,516 (23.8%)	2,111 (23.0%)	405 (29.0%)	
Others	1,537 (14.5%)	1,369 (14.9%)	168 (12.0%)	
NA	428 (4.0%)	423 (4.6%)	5 (0.4%)	
Histology type (%)				<0.001
Carcinoid tumor	9,560 (90.4%)	8,677 (94.5%)	883 (63.2%)	
Neuroendocrine carcinoma	1,004 (9.5%)	503 (5.5%)	501 (35.9%)	
Atypical carcinoid tumor	16 (0.2%)	3 (0.0%)	13 (0.9%)	
Surgical treatment (%)				<0.001
No surgery	2,106 (19.9%)	1,573 (17.1%)	533 (38.2%)	
Tumor destruction	34 (0.3%)	30 (0.3%)	4 (0.3%)	
Tumor resection	8,306 (78.5%)	7,467 (81.3%)	839 (60.1%)	
Surgery, unknown type	25 (0.2%)	19 (0.2%)	6 (0.4%)	
NA	109 (1.0%)	94 (1.0%)	15 (1.1%)	
Tumor size (cm)				<0.001
<1	3,119 (29.5%)	2,889 (31.5%)	230 (16.5%)	
1–2	645 (6.1%)	539 (5.9%)	106 (7.6%)	
>2	477 (4.5%)	198 (2.2%)	279 (20.0%)	
NA	6,339 (59.9%)	5,557 (60.5%)	782 (56.0%)	
Tumor numbers	1.00 (1.00–1.00)	1.00 (1.00–1.00)	1.00 (1.00–2.00)	<0.001
Regional lymph nodes invasion (%)				<0.001
Negative	10,079 (95.3%)	8,835 (96.2%)	1,244 (89.0%)	
Positive	193 (1.8%)	88 (1.0%)	105 (7.5%)	
NA	308 (2.9%)	260 (2.8%)	48 (3.4%)	
Grade (%)				<0.001
Well differentiated	1,783 (16.9%)	1,559 (17.0%)	224 (16.0%)	
Moderately differentiated	354 (3.3%)	287 (3.1%)	67 (4.8%)	
Poorly differentiated	266 (2.5%)	36 (0.4%)	230 (16.5%)	
Undifferentiated	111 (1.0%)	12 (0.1%)	99 (7.1%)	
NA	8,066 (76.2%)	7,289 (79.4%)	777 (55.6%)	
TNM staging (%)				<0.001
I	1,417 (13.4%)	1,271 (13.8%)	146 (10.5%)	
II	128 (1.2%)	99 (1.1%)	29 (2.1%)	
III	67 (0.6%)	27 (0.3%)	40 (2.9%)	
IV	185 (1.7%)	16 (0.2%)	169 (12.1%)	
NA	8,783 (83.0%)	7,770 (84.6%)	1,013 (72.5%)	
Summary staging (%)				<0.001
*In situ*	39 (0.4%)	37 (0.4%)	2 (0.1%)	
Localized	8,648 (81.7%)	7,897 (86.0%)	751 (53.8%)	
Regional	205 (1.9%)	97 (1.1%)	108 (7.7%)	
Distant	447 (4.2%)	59 (0.6%)	388 (27.8%)	
NA	1,241 (11.7%)	1,093 (11.9%)	148 (10.6%)	
Total	10,580	9,183	1,397	

*NA refers to the count of missing values*.

### Survival Factors Associated With the Prognosis With R-NETs

In the cohort of SEER database patients, multivariable Cox regression analysis showed that age, gender, race, histologic type, tumor size, tumor number, summary stage, and surgical treatment were independent prognostic factors for the 5-year survival status (Table 2). It was found that older age, increased tumor grade, larger tumor size, more tumor numbers, and advanced staging could significantly decrease the 5-year survival. Also, histologic type of atypical carcinoid tumor was associated with a worse survival outcome than the histologic type of carcinoid tumor and neuroendocrine carcinoma. The female gender was a protective factor for the 5-year survival status.

**Table 2:** Multivariable Cox regression results.

**Characteristics**	**B**	***P* value**	**HR value**	**HR 95%CI**
Female	0.359	<0.001	0.699	0.643–0.760
Race—White		<0.001		
Black	0.371	<0.001	1.449	1.320–1.592
Other	0.180	0.006	0.835	0.733–0.950
NA	1.787	<0.001	0.167	0.087–0.323
Histologic type—carcinoid tumor		<0.001		
Neuroendocrine carcinoma	0.482	<0.001	1.619	1.378–1.901
Atypical carcinoid tumor	1.334	<0.001	3.796	2.168–6.645
Grade—well differentiated		<0.001		
Moderately differentiated	0.106	0.378	1.112	0.879–1.406
Poorly differentiated	0.798	<0.001	2.221	1.802–2.738
Undifferentiated	0.800	<0.001	2.225	1.714–2.888
NA	0.059	0.383	0.942	0.825–1.077
Summary stage—*In situ*		<0.001		
Localized	0.089	0.828	1.093	0.490–2.438
Regional	1.153	0.006	3.169	1.386–7.246
Distant	2.109	<0.001	8.241	3.641–18.655
NA	0.073	0.861	1.075	0.477–2.423
Surgical approach—no surgery		<0.001		
Tumor destruction	0.333	0.326	0.717	0.369–1.393
Unknown type	0.309	<0.001	0.734	0.661–0.815
Tumor resection	0.142	0.674	1.153	0.594–2.237
NA	0.048	0.820	0.954	0.633–1.436
Tumor size <1 cm		0.022		
1–2 cm	0.250	0.009	1.284	1.066–1.546
>2 cm	0.249	0.011	1.283	1.059–1.554
NA	0.104	0.084	1.110	0.986–1.248
Tumor numbers	0.358	<0.001	1.431	1.359–1.507
Age	0.058	<0.001	1.060	1.056–1.064

*NA refers to the count of missing values*.

*HR, hazard rate*.

### Feature Analysis

**Figure 2:**
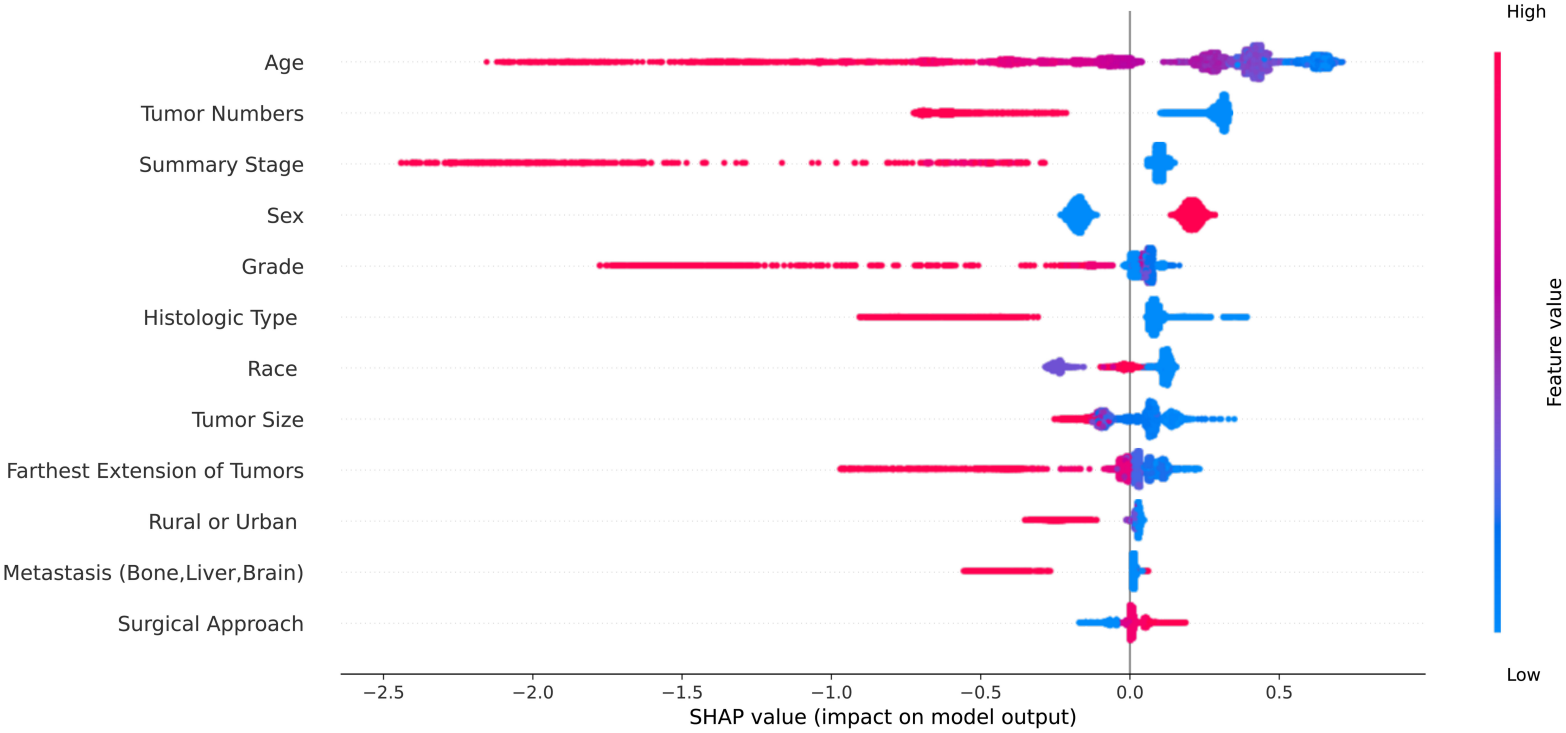
The Shapley additive explanations summary plot of the predictive model ordered 12 features based on their impact on the 5-year survival status. The red dot represents the high value of the feature and the blue dot represents the low value of the feature.

In the cohort of SEER database patients, we evaluated all machine learning predictive models using the SHAP method, and finally, we selected 12 features that were considered important features in all machine learning predictive models (Table 3). The SHAP summary plot of the predictive model ordered 12 features based on their impact on the 5-year survival status in Figure 2. The higher SHAP value of a feature indicates the greater possibility of a 5-year survival. The color of the dot represents a large or small feature value. Red indicates that the feature value is large, purple indicates that the feature value is close to the overall average, and blue indicates that the feature value is small. Take age as an example, we found that older age was associated with the lower likelihood of 5-year survival.

**Table 3:** Features used in machine learning predictive models.

**General information**	
	Age (years)
	Gender
	Race
	Rural or urban
**Tumor information**	
	Histologic type
	Grade
	Summary stage
	Surgical approach
	Tumor size (mm)
	Metastasis (Bone, Liver, Brain)
	Farthest extension of tumors (mm)
	Tumor numbers

### Model Evaluation

The SEER database started recording the seventh edition of the AJCC staging system for R-NETs in 2010. To further evaluate the predictive performance of the AJCC seventh staging system and the machine learning predictive model, we chose patients from the SEER database during the 2000–2009 period as the training set, involving a total of 7,380 patients. Based on the data from 2010 to 2017, we excluded patients without the AJCC seventh staging. Finally, a total of 1,797 patients were selected in the internal validation set (Figure 1).

**Figure 3:**
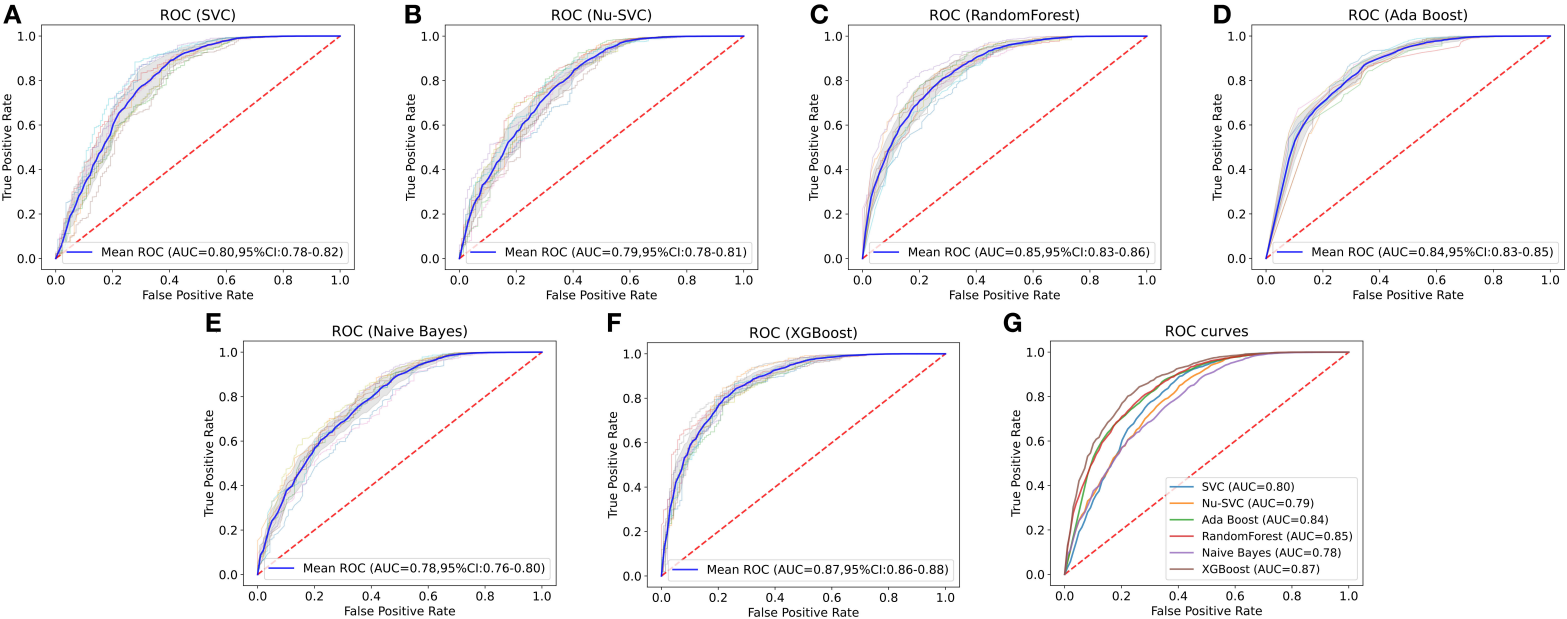
Comparison of the AUCs of six machine learning algorithms. The predictive model using the eXtreme Gradient Boosting (XGBoost) algorithm had the best predictive performance. SVC, C-Support Vector Classification; RF, random forest; NB, Naive Bayes.

Clinical characteristics of the training set and internal validation set were summarized in Table 4. First, we used the training set to establish and train machine learning models. After parameters adjustment and algorithms comparison, the AUC values of six machine learning models were higher than 0.78, thereby demonstrating a good predictive ability of predictive models (Figure 3). As described in Table 4, the AUC of SVC was 0.80 (95%CI: 0.78–0.82), the AUC of Nu-SVC was 0.79 (95%CI: 0.78–0.81), the AUC of RF was 0.85 (95%CI: 0.83–0.86), the AUC of Ada Boost was 0.84 (95%CI: 0.83–0.85), the AUC of NB was 0.78 (95%CI: 0.76–0.80), and the AUC of XGBoost was 0.87 (95%CI: 0.86–0.88). Among them, the predictive model using the XGBoost algorithm had the best predictive performance. Subsequently, we compared the XGBoost model and the AJCC seventh staging system for predicting the 5-year survival status in R-NETs.

**Table 4:** Main characteristics of training set and internal validation set.

**Variable**	**Training set**	**Internal validation set**	***P* value**
Age (years)	56.1 ± 12.0	56.6 ± 11.8	0.107
Gender (%)			0.969
Male	3,663 (49.6%)	891 (49.6%)	
Female	3,717 (50.4%)	906 (50.4%)	
Race (%)			0.001
White	4,381 (59.4%)	1,001 (55.7%)	
Black	1,688 (22.9%)	442 (24.6%)	
Others	1,043 (14.1%)	305 (17.0%)	
NA	268 (3.6%)	49 (2.7%)	
Histology type (%)			<0.001
Carcinoid tumor	6,964 (94.4%)	1,391 (77.4%)	
Neuroendocrine carcinoma	409 (5.5%)	402 (22.4%)	
Atypical carcinoid tumor	7 (0.1%)	4 (0.2%)	
Surgical treatment (%)			<0.001
No surgery	1,385 (18.8%)	262 (14.6%)	
Tumor destruction	29 (0.4%)	2 (0.1%)	
Tumor resection	5,885 (79.7%)	1,522 (84.7%)	
Surgery, unknown type	17 (0.2%)	5 (0.3%)	
NA	64 (0.9%)	6 (0.3%)	
Tumor size (cm)			<0.001
<1	1,754 (23.8%)	1,251 (69.6%)	
1~2	388 (5.3%)	227 (12.6%)	
>2	215 (2.9%)	218 (12.1%)	
NA	5,023 (68.1%)	101 (5.6%)	
Tumor numbers	1.00 (1.00–1.00)	1.00 (1.00–1.00)	0.135
Regional lymph nodes invasion (%)			<0.001
Negative	7,051 (95.5%)	1,726 (96.0%)	
Positive	130 (1.8%)	60 (3.3%)	
NA	199 (2.7%)	11 (0.6%)	
Grade (%)			<0.001
Well differentiated	720 (9.8%)	667 (37.1%)	
Moderately differentiated	164 (2.2%)	120 (6.7%)	
Poorly differentiated	125 (1.7%)	108 (6.0%)	
Undifferentiated	48 (0.7%)	47 (2.6%)	
NA	6,323 (85.7%)	855 (47.6%)	
TNM staging (%)			–
I	0 (0.0%)	1,417 (78.9%)	
II	0 (0.0%)	128 (7.1%)	
III	0 (0.0%)	67 (3.7%)	
IV	0 (0.0%)	185 (10.3%)	
NA	7,380 (100.0%)	0 (0.0%)	
Summary staging (%)			<0.001
*In situ*	30 (0.4%)	0 (0.0%)	
Localized	6,130 (83.1%)	1,543 (85.9%)	
Regional	125 (1.7%)	66 (3.7%)	
Distant	223 (3.0%)	188 (10.5%)	
NA	872 (11.8%)	0 (0.0%)	
5–Year survival	6,618 (89.7%)	1,413 (78.6%)	<0.001
Total	7,380	1,797	

*NA refers to the count of missing values*.

#### Internal Validation

**Figure 4:**
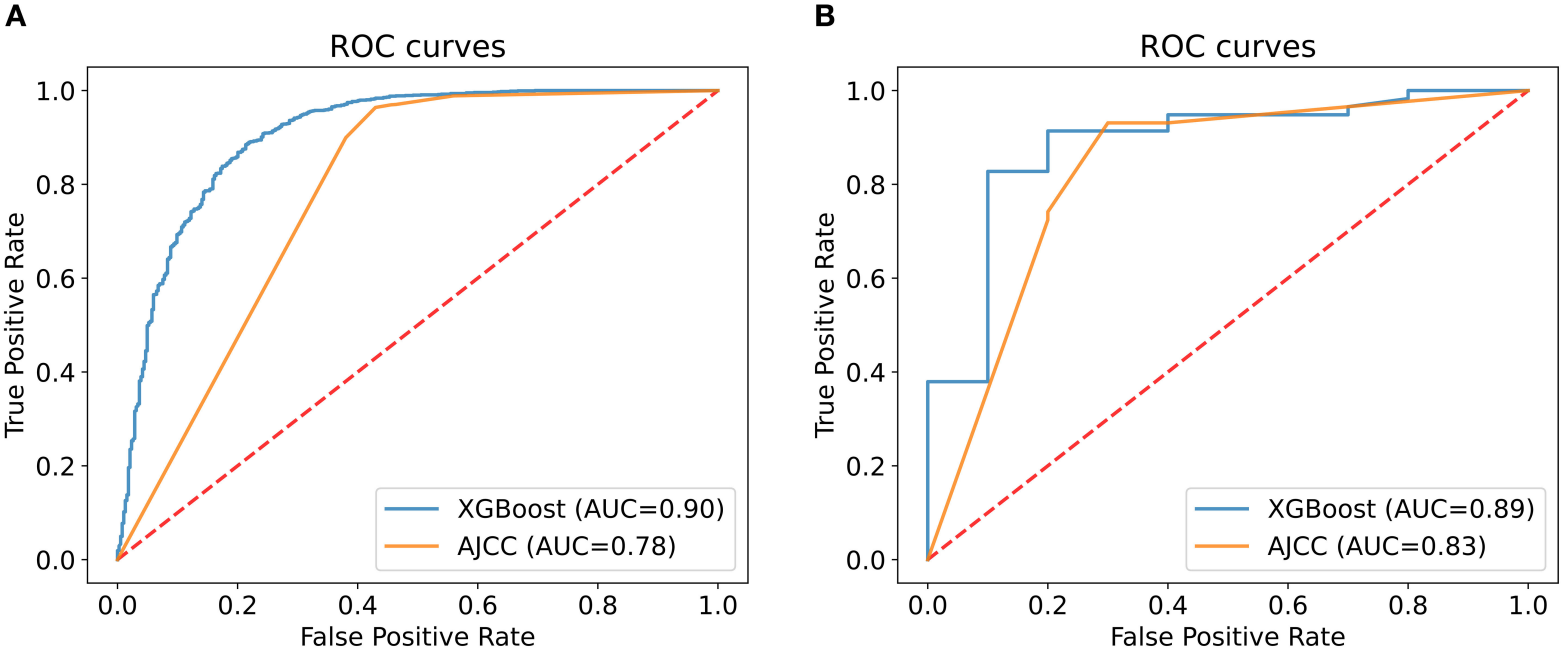
Comparison of the XGBoost model and the AJCC seventh staging system. The area under the curve (AUC) of the XGBoost model to predict 5-year survival status was larger than that of the AJCC seventh staging system in both internal **(A)** and external validation **(B)**.

We analyzed the predictive performance of the machine learning predictive model and the AJCC seventh staging system in the internal validation set. We calibrated the machine learning model using the parametric approach based on Platt's logistic model, the Brier score obtained from the machine learning predictive model was 0.084, indicating good calibration and discriminative ability. The ROC curve of machine learning predictive models (AUC = 0.90, 95% CI: 0.89–0.92) was better than the ROC curve of AJCC seventh staging system (AUC = 0.78, 95% CI: 0.76–0.80). The sensitivity of machine learning models was not inferior to the traditional AJCC staging system, and a high specificity was also observed (Figure 4; Table 5). Compared with the AJCC seventh staging system, more patients were correctly classified by machine learning predictive models (NRI = 0.151, 95%CI: 0.103–0.199, *P* < 0.001).

**Table 5:** Performance of machine learning predictive models and the AJCC seventh staging system.

**Predictive models**	**AUC**	**95% CI of AUC**
**Machine learning predictive models**		
SVC	0.80	0.78–0.82
Nu–SVC	0.79	0.78–0.81
Random Forest	0.85	0.83–0.86
Ada Boost	0.84	0.83–0.85
Naïve Bayes	0.78	0.76–0.80
XGBoost	0.87	0.86–0.88
**Internal validation**		
Machine Learning (XGBoost)	0.90	0.89–0.92
AJCC seventh staging system	0.78	0.76–0.80
**External validation**		
Machine Learning (XGBoost)	0.89	0.77–1.00
AJCC seventh staging system	0.83	0.66–0.99

*AJCC, American Joint Committee on Cancer; AUC, area under the curve; SVC, support vector classification; XGBoost, eXtreme gradient boosting*.

#### External Validation

To evaluate the performance of machine learning predictive models, we collected data of 68 patients with R-NETs from Peking Union Medical College Hospital as the external validation set. The basic characteristics of 68 patients are summarized in Table 6. In external validation, the machine learning predictive model had a better degree of calibration and its Brier score is 0.080. Although the number of data was relatively small, the ROC curve of the machine learning predictive model (AUC = 0.89, 95%CI: 0.77–1.00) was still superior to the ROC curve of AJCC seventh staging system (AUC = 0.83, 95% CI: 0.66–0.99) (Figure 4; Table 5). In addition, more patients were correctly classified by the machine learning predictive model (NRI = 0.190, 95%CI: 0.089–0.291, *P* < 0.001).

**Table 6:** Main characteristics of SEER database and China database.

**Variable**	**SEER database**	**China database**	***P*-value**
Age (years)	56.3 ± 11.9	49.3 ± 11.9	<0.001
Gender (%)			0.447
Male	5,267 (49.8%)	37 (54.4%)	
Female	5,313 (50.2%)	31 (45.6%)	
Race (%)			<0.001
White	6,099 (57.6%)	0 (0.0%)	
Black	2,516 (23.8%)	0 (0.0%)	
Others	1,537 (14.5%)	68 (100.0%)	
NA	428 (4.0%)	0 (0.0%)	
Histology type (%)			0.329
Carcinoid tumor	9,560 (90.4%)	58 (85.3%)	
Neuroendocrine carcinoma	1,004 (9.5%)	10 (14.7%)	
Atypical carcinoid tumor	16 (0.2%)	0 (0.0%)	
Surgical treatment (%)			0.086
No surgery	2,106 (19.9%)	5 (7.4%)	
Tumor destruction	34 (0.3%)	0 (0.0%)	
Surgery, unknown type	8,306 (78.5%)	63 (92.6%)	
Tumor resection	25 (0.2%)	0 (0.0%)	
NA	109 (1.0%)	0 (0.0%)	
Tumor size (cm)			<0.001
<1	3,119 (29.5%)	37 (54.4%)	
1–2	645 (6.1%)	23 (33.8%)	
>2	477 (4.5%)	8 (11.8%)	
NA	6,339 (59.9%)	0 (0.0%)	
Tumor numbers	1.00 (1.00–1.00)	1.00 (1.00–1.00)	–
Regional lymph nodes invasion (%)			<0.001
Negative	10,079 (95.3%)	54 (79.4%)	
Positive	193 (1.8%)	14 (20.6%)	
NA	308 (2.9%)	0 (0.0%)	
Grade (%)			<0.001
Well differentiated	1,783 (16.9%)	50 (73.5%)	
Moderately differentiated	354 (3.3%)	16 (23.5%)	
Poorly differentiated	266 (2.5%)	2 (2.9%)	
Undifferentiated	111 (1.0%)	0 (0.0%)	
NA	8,066 (76.2%)	0 (0.0%)	
TNM staging (%)			<0.001
I	1,417 (13.4%)	44 (64.7%)	
II	128 (1.2%)	1 (1.5%)	
III	67 (0.6%)	13 (19.1%)	
IV	185 (1.7%)	10 (14.7%)	
NA	8,783 (83.0%)	0 (0.0%)	
Summary staging (%)			<0.001
*In situ*	39 (0.4%)	6 (8.88%)	
Localized	8,648 (81.7%)	47 (69.1%)	
Regional	205 (1.9%)	4 (5.9%)	
Distant	447 (4.2%)	11 (16.2%)	
NA	1,241 (11.7%)	0 (0.0%)	
5-Year survival	9,183 (86.8%)	58 (85.3%)	0.715
Total	10,580	68	

*NA refers to the count of missing values*.

## Discussion

In this study, we constructed prognostic models through six common machine learning algorithms based on clinical features. To our knowledge, this is the first study to use machine learning algorithms predicting the survival status of patients with R-NETs. As the result showed, these models had a good predictive ability based on a large number of patient data from the SEER database. Our study proved that machine learning predictive models performed better than the AJCC staging system. In both internal validation and external validation, the XGBoost model performed best among all the models overall.

Machine learning can discern patterns from large datasets. Identified patterns are then used to encode a mathematical model, which applies to new data for further validation (10). Applications of machine learning in medicine are being used in disease diagnosis, prognosis, therapy development, and treatment assessment. As the number of data grows, machine learning algorithms will develop more accurate predictive power. Because NETs are relatively rare tumors, only a few machine learning applications studies have focused on NETs. Most research studies have focused on disease diagnosis, such as imaging parameters (11), pathological manifestations (12), or biomarker analysis (13). The SEER registry database effectively compensates for the deficiencies in the clinical data in traditional research centers. Using the SEER database from 1973 to 2014, research has found that the models developed with classic machine learning algorithms performed well in survival prediction of pancreas neuroendocrine tumors (14). At present, there is no relevant literature report about machine learning prognostic models of patients with R-NETs.

A gradual increase of patients with R-NETs has highlighted the need for a more comprehensive and refined system for disease prognosis. To date, a variety of prognostic predictive systems have been established. In 2008, a study developed a new TNM staging system through data from the SEER database. Primary tumor size, depth of invasion, lymph node involvement, and distant metastasis were related to the prognosis of R-NETs (15). Fields et al. indicated that the number of positive locoregional lymph nodes was an independent factor to estimate survival of R-NETs (16). In another recent study by Capurso and colleagues, the prognostic role of the ENETs staging and grading systems was evaluated in rectal neuroendocrine neoplasms. They reported that the presence of metastatic disease at diagnosis and the proliferative index was associated with overall and progression-free survival (6). Feng and collaborators developed a nomogram predicting the overall survival of R-NETs. They found that age, sex, tumor size, and TNM stage were independently correlated with prognosis (17). Apart from these, we found out that tumor numbers, race, and surgical approach also influenced prognosis.

Prognostic risk models based on a single anatomical stage or pathological grade have some limitations. They mainly relate to the depth of tumor invasion, tumor size, involvement of lymph nodes, the presence of metastatic disease, and the Ki-67 index. The machine learning model based on multiple factors is expected to be a more effective tool in predicting the prognosis. Machine learning can process a large number of data in a short time, and it has certain advantages in comparison with the traditional methods. Currently, logistic regression is the most frequently used traditional analytical statistical algorithm, which can determine risk predictive factors in the short term (18). However, clinical characteristics often have a non-linear relationship, which makes logistic regression sometimes fail to obtain the desired results. Machine learning can use algorithms and statistical models to identify data and learn from the data, which can handle non-linear data (19). Therefore, many researchers support the use of more advanced machine learning algorithms to build predictive models for big data analysis. In most studies, the performance of machine learning models is better than that of logistic regression.

The challenges of building machine learning models include multicollinearity, incorrect imputation, data leakage, neglecting feature scaling, and normalization, which will cause overfitting, loss of feature importance interpretability, and model instability. Using a clear validation set, feature importance analysis, and standard techniques provides efficient approaches for establishing prognostic models (20). As shown in Table 1, some information was missing in the database. We used the k-Nearest Neighbors approach to avoid incorrect imputation. During the validation process, we used the algorithm to automatically split the data into a training set and a validation set, thus ensuring a clean validation set.

The ultimate purpose of constructing predictive models is to facilitate clinical decision making. We built an accessible online application (https://gastrointestinal.github.io/NET/) based on the XGBoost algorithm for the convenience of clinical practice.

Despite its merits, this study has certain limitations. First, this was a retrospective study, so the potential for selective bias was inevitable. Second, because R-NETs were a rare disease in China, the sample size of external validation is relatively small. Although machine learning models showed good predictive performance with the currently small dataset, they require a larger scale study for external validation in the future. Third, we cannot analyze the appropriateness of medication due to the lack of detailed drug regimens in the SEER database. Finally, machine learning predictive models do not provide a predictive scoring system. This limits their applications in routine clinical practice. However, the online application we have established could be more conveniently combined with the electronic medical record system to help clinical decision making.

In summary, we explored and analyzed the demographic characteristics of patients with R-NETs and used machine learning algorithms to establish survival predictive models. The XGBoost algorithm had better predictive power than the AJCC staging system, which had a potential clinical application value.

## Data Availability Statement

The datasets presented in this study can be found in online repositories. The names of the repository/repositories and accession number(s) can be found below: Data of patients with R-NETs were extracted from the SEER database (2000–2017), using the SEER^*^ Stat software version 8.3.6.1. (https://staging.seer.cancer.gov).

## Author Contributions

JingL: designed the study. KL,TX, and JinL: material preparation, data collection, and analysis. XC: wrote the manuscript. All authors commented on previous versions of the manuscript, read, and approved the final manuscript.

## Funding

This study was supported by the National Natural Science Foundation of China (81770559) and the Chinese Academy of Medical Sciences (CAMS) Initiative for Innovative Medicine (CAMS-2016-I2M-1-007).

## Conflict of Interest

The authors declare that the research was conducted in the absence of any commercial or financial relationships that could be construed as a potential conflict of interest.

## Publisher's Note

All claims expressed in this article are solely those of the authors and do not necessarily represent those of their affiliated organizations, or those of the publisher, the editors and the reviewers. Any product that may be evaluated in this article, or claim that may be made by its manufacturer, is not guaranteed or endorsed by the publisher.

## References

[1] Caplin M Sundin A Nillson O Baum RP Klose KJ Kelestimur F. ENETS Consensus Guidelines for the management of patients with digestive neuroendocrine neoplasms: colorectal neuroendocrine neoplasms. Neuroendocrinology. (2012) 95:88–97. 10.1159/000335594 22261972

[2] McDermott FD Heeney A Courtney D Mohan H Winter D. Rectal carcinoids: a systematic review. Surg Endosc. (2014) 28:2020–6. 10.1007/s00464-014-3430-0 24584484

[3] Yao JC Hassan M Phan A Dagohoy C Leary C Mares JE. One hundred years after “carcinoid”: epidemiology of and prognostic factors for neuroendocrine tumors in 35,825 cases in the United States. J Clin Oncol. (2008) 26:3063–72. 10.1200/JCO.2007.15.4377 18565894

[4] Amin MB Edge SB. AJCC Cancer Staging Manual. New York, NY: Springer (2017).

[5] Bosman FT Carneiro F Hruban RH Theise ND. WHO classification of tumours of the digestive system. Geneva: World Health Organization (2010).

[6] Capurso G Gaujoux S Pescatori LC Panzuto F Panis Y Pilozzi E. The ENETS TNM staging and grading system accurately predict prognosis in patients with rectal NENs. Dig Liver Dis. (2019) 51:1725–30. 10.1016/j.dld.2019.07.011 31405587

[7] Troyanskaya O Cantor M Sherlock G Brown P Hastie T Tibshirani R. Missing value estimation methods for DNA microarrays. Bioinformatics. (2001) 17:520–5. 10.1093/bioinformatics/17.6.520 11395428

[8] Pedregosa F Varoquaux G Gramfort A Michel V Thirion B Grisel O. Scikit-learn: machine learning in python. J Mach Learn Res. (2011) 12:2825–30.

[9] Lundberg SM Lee SI. A unified approach to interpreting model predictions. Adv Neur In. (2017) 17–30.

[10] Scott IA. Demystifying machine learning—a primer for physicians. Intern Med J. (2021) 1–18. 10.1111/imj.15200 33462882

[11] Zhang T Zhang Y Liu X Xu H Chen C Zhou X. Application of radiomics analysis based on CT combined with machine learning in diagnostic of pancreatic neuroendocrine tumors patient's pathological grades. Front Oncol. (2020) 10:521831. 10.3389/fonc.2020.521831 33643890 PMC7905094

[12] Govind D Jen KY Matsukuma K Gao G Olson KA Gui D. Improving the accuracy of gastrointestinal neuroendocrine tumor grading with deep learning. Sci Rep. (2020) 10:11064. 10.1038/s41598-020-67880-z 32632119 PMC7338406

[13] Kjellman M Knigge U Welin S Thiis-Evensen E Gronbaek H Schalin-Jantti C. A plasma protein biomarker strategy for detection of small intestinal neuroendocrine tumors. Neuroendocrinology. (2020) 30:20. 10.1159/000510483 32721955 PMC8686712

[14] Song Y Gao S Tan W Qiu Z Zhou H Zhao Y. Multiple machine learnings revealed similar predictive accuracy for prognosis of PNETs from the surveillance, epidemiology, and end result database. J Cancer. (2018) 9:3971–8. 10.7150/jca.26649 30410601 PMC6218767

[15] Landry CS Brock G Scoggins CR McMasters KM Martin RC. A proposed staging system for rectal carcinoid tumors based on an analysis of 4701 patients. Surgery. (2008) 144:460–6. 10.1016/j.surg.2008.05.005 18707046

[16] Fields AC McCarty JC Ma-Pak L Lu P Irani J Goldberg JE. New lymph node staging for rectal neuroendocrine tumors. J Surg Oncol. (2019) 119:156–62. 10.1002/jso.25307 30481376

[17] Feng X Wei G Wang W Zhang Y Zeng Y Chen M. Nomogram for individually predicting overall survival in rectal neuroendocrine tumours. BMC Cancer. (2020) 20:865. 10.1186/s12885-020-07328-9 32907602 PMC7488006

[18] Collins GS Reitsma JB Altman DG Moons KG. Transparent reporting of a multivariable prediction model for individual prognosis or diagnosis (TRIPOD): the TRIPOD statement. Ann Intern Med. (2015) 162:55–63. 10.7326/M14-0697 25560714

[19] Hamet P Tremblay J. Artificial intelligence in medicine. Metabolism. (2017) 69S:S36–S40. 10.1016/j.metabol.2017.01.011 28126242

[20] Gilvary C Madhukar N Elkhader J Elemento O. The missing pieces of artificial intelligence in medicine. Trends Pharmacol Sci. (2019) 40:555–64. 10.1016/j.tips.2019.06.001 31277839

